# Research on Intelligent English Education Based on the Short Video Recommendation Algorithm

**DOI:** 10.1155/2023/2317589

**Published:** 2023-07-07

**Authors:** Xiaoyu Li

**Affiliations:** School of Foreign Language, Jilin University of Architecture and Technology, Changchun 130111, China

## Abstract

In order to solve the problems of English education in the form of a short video, a research method of English intelligent education based on a short video recommendation algorithm was proposed. The recommendation system is a branch of artificial intelligence data mining, which improves the efficiency of short videos for English learning. The density ratio of users and video scoring matrix was 1000000/(1030 × 9394) = 10.3%. The dataset was a relatively sparse matrix. The original dataset was randomly divided into the training set and the test set, accounting for 80% and 20%, respectively. Then, the results of the short video recommendation algorithm were elaborated based on time weighting. Finally, the intelligent initial question bank of English intelligent education based on a short video recommendation algorithm was elaborated, which provided a guarantee for the promotion of short videos in English education.

## 1. Introduction

With the further deepening of China's reform and by opening up the everchanging demand for talents, the Ministry of Education has gradually begun to pay attention to the use of applied foreign language testing methods and the cultivation of talents. The essence of education is to cultivate talents and improve the quality of the people, and English wisdom education is more focused on facing the future. The challenge of smart English education to frontline teachers is not only limited to creativity and expressiveness but also needs to keep pace with the times and be able to skillfully use some advanced concepts such as computer technology and cloud technology to carry out scientific teaching design and big data analysis, so as to effectively improve classroom effectiveness and the quality of student learning. In the information age of big data, teachers should be able to make full use of Internet data, find needed information, put forward creative topics, draw conclusions through analysis and comparison, or find the crux of problems and solutions, eliminate pain points and difficulties, and pay attention to the timeliness of data and reliability, etc. The definition of English wisdom education is as follows: English wisdom education is the informatization of English education, which refers to the comprehensive and in-depth application of modern information technology in the field of education (education management, education teaching, and education research) to promote the process of education reform and development. Its main technical features are digitization, networking, intelligence, and multimedia; its basic features are openness, sharing, interaction, collaboration, and ubiquity. That is, to promote the modernization of education with educational informatization and the use of information technology to change the traditional model.

E-commerce companies such as Amazon and Taobao are the first Internet products to use algorithmic protocols. They can filter out products related to consumer goods by analyzing consumer purchases, so as to improve the purchase rate of users, and the ability of enterprises to develop new products and the cycle of enterprises developing new products. One of the characteristics of the Web 2.0 era is the low start of content production. UGC has become a key element in manufacturing. Conventional algorithms have been gradually applied to content production platforms, such as short video apps Tiktok and Kuaishou. A recommendation algorithm is a kind of algorithm in computer science which uses some mathematical algorithms to determine what users may like. At present, the good application of recommendation algorithm is mainly the network. Due to the wide variety of UGCs, it is also important to fully customize the personalization for the user. However, in the process of iteration, the algorithmic recommendation technology still needs to break through many bottlenecks. Moreover, recommendations that do not meet user needs will affect users' experience. A short video recommendation algorithm has a great influence on people. However, the influence should not only be applied in the business field but also in academic and educational fields, so as to give full play to the advantages of short video recommendation algorithms and to promote the development of English intelligent education.

## 2. Literature Review

Dlamini et al. believed that the high compatibility of 5G network communication technology and the flat IP network provided sufficient technical support and new development opportunities for short video [1]. Wu, X. et al believed that “browsing short videos for a while excitement and browsing continuously for a continuous excitement” was a very common experience when using short video apps. This immersive reading experience was also known as the “flow experience”. The flow theory was first proposed by psychologist Csikszentmihalyi of The University of Chicago, also known as an “immersive experience” [2].

Williams believed that according to the financial industry analysis report, the user penetration rate of short video reached 70% and it became the third traffic entrance of the Internet. A short video would continue to occupy users' time [3]. Karney and Bradbury believed that information distribution based on an intelligent algorithm recommendation system was a very important link in the formation of the recommendation flow of short video apps. The system mainly combined multiple types of recommendation algorithms to present content recommendation flow to users, which shaped and strengthened users' sense of immersion [4]. Huang and Fu believed that it could not only meet users' existing information needs but was also able to mine users' potential information needs through modeling [5]. Wu believed that the platform recommended short video content in line with the personal interests and hobbies of users to enhance users' loyalty and satisfaction. It could reflect the features of artificial intelligence that are based on the historical record of interaction with users, and the recommendation algorithm could better grasp the potential value needs of users, predict the content that users may like, and provide users with their own recommendation flow [6].

**Figure 1:**
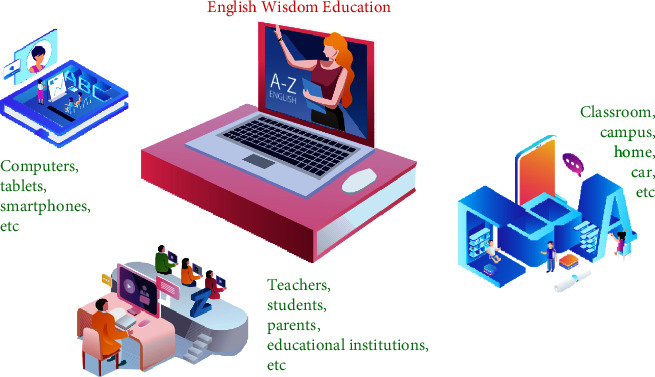
Research on English intelligent education.

Zhang believes that a convolutional neural network is a feedforward neural network with a deep structure including convolutional computation, and it is one of the representative algorithms of deep learning [7]. Thiem argued that convolutional neural networks can represent learning and can access different interpretations according to their hierarchical shared information, hence the term “translation-invariant artificial neural networks” [8]. Hasudungan believes that convolutional neural networks follow the development of visual impairment and can achieve supervised and unsupervised learning. The integration of convolution kernels in hidden layers and the sparsity of interlayer connections enables convolutional neural networks to characterize points with a small amount of computation [9]. Kim believed that the learning of pixel and audio, for example, had stable effects with no additional feature engineering requirements for data [10]. The main contents of intelligent education are shown in Figure 1.

## 3. Research Methods

### 3.1. Related Technologies and Theories of Short Video Recommendation Algorithms

#### 3.1.1. Convolutional Neural Network

The convolutional operator is an important processing operator in the convolutional layer. The convolutional operator slides the kernel during operation. The calculation formula of the convolutional operator is shown in following formula.

(1) $$\begin{array}{c} x_{j}^{i}=f\left(\underset{ieMj}{\sum }x_{j}^{l-1}\times Kernel_{ij}^{l}+b_{j}^{l}\right)\text{,}\\ x_{i}^{l}=f\left(feature\left(i,j\right)\right)\text{,} \end{array}$$

where *x* is the result and the output feature obtained by convolution at the *l* layer and *f* represents the activation function. Kernel represents convolutional kernel matrix and *b* represents bias. *M* represents the number of feature inputs.

**Figure 2:**
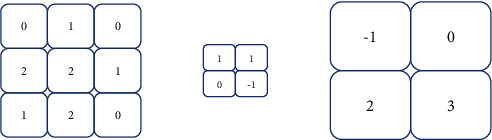
Maximum pooling of convolutional neural networks.

**Figure 3:**
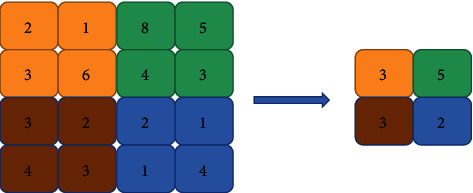
Average pooling of convolutional neural networks.

The pooling layer of the convolutional neural network is the further processing of the image, which reduces its features again and has the effect of reducing overfitting. The commonly used pooling methods of the pooling layer mainly include maximum pooling and average pooling [11, 12]. The input of a convolutional neural network is usually two-dimensional. Its main application is to process images and videos, especially in the field of visual recognition and image classification. A convolutional neural network imitation of biological visual perception (visual perception) mechanism construction can be supervised learning and unsupervised learning. A two-dimensional convolutional neural network occupies a certain proportion. Maximum pooling and average pooling are shown in Figures 2 and 3.

#### 3.1.2. Recursive Neural Network

**Figure 4:**
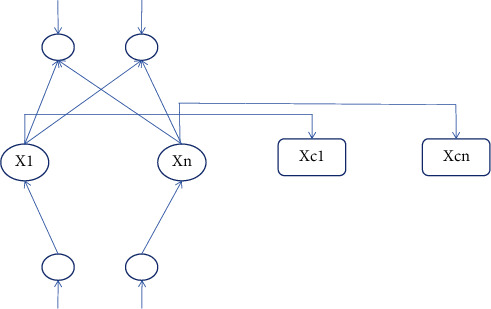
Structure of a recursive neural network.

A recursive neural network is a kind of deformation of the BP network. It adds a continuity layer and a memory unit module to its network structure so as to enhance the stability of the network and enable it to change features at the appropriate time. Many companies have highlighted cybersecurity priorities in digital strategy, and CISO's awareness of the crisis is spreading security responsibility across the organization and working to change the IT culture. Because it comes from a BP network with a timing sequence, it has a certain dependence on time [13]. Its main feature is the current state and output. The last time point of the state and the input determines the current state and output. The interval of the choice of time steps in the recursive neural network is not too long, which will lead to an increase in the probability of convergence problems. If the two sentences mean a similar meaning; then, the two vectors that encode each other will be close apart, and if the two sentences mean a very different meaning; then, the encoded posterior vector will be far apart. So more appropriate activation function needs to be chosen to reduce the convergence phenomenon. The input value of a recursive neural network is *u*(*t* − 1) and the output value is *y*(*t*). The structure of a recursive neural network is shown in Figure 4.

#### 3.1.3. Local Linear Embedding Algorithm

A local linear embedding algorithm is a local geometry structure preservation algorithm. The basic idea is to assume that each sample point can find *k* sample points close to it in its local domain; then, this sample point can be linearly represented by *k* sample points, and then the data are mapped from high-dimensional complex space to a low-dimensional space. Let the set of given eigenvectors be *X*={*x*_1_, *x*2,…, *x*_*n*_}, *x*_*i*_ ∈ RD. *N* represents the number of feature vectors, and the output vector *Y*={*y*_1_, *y*_2_,…, *y*_*n*_}*y*_*i*_ ∈ *R*_*d*_, *d* << *D* can be obtained after dimensionality reduction by a local linear embedding algorithm.

For the selection of field size *k, k* feature vectors closest to each feature vector *ix* is selected as the *k* nearest neighbor points of sample point *ix*. Then, the reconstruction weight matrix is calculated. For each feature vector *ix*, we calculate the nearest neighbor weight matrix obtained from *k* nearest neighbor points and define the error function *ε* (*w*). The local linear embedding algorithm formula can be represented as follows:

(2) $$\begin{array}{c} \varepsilon \left(w\right)={\overset{N}{\underset{i=1}{\sum }}W_{i}^{T}\left(xi-xj\right)\left(xi-xj\right)}^{T}Wi\text{.} \end{array}$$

In the formula, *x*_*i*_(*j*=1, 2,…, *k*) is the *k* nearest neighbor points of *x*_*i*_. *w*_*ij*_(*i*=1, 2,…, *N*;  *j*=1, 2,…, *k*) is the weight between *x*_*i*_ and *x*_*j*_ and it satisfies the following formula.

(3) $$\begin{array}{c} \overset{k}{\underset{j=1}{\sum }}wij=1. \end{array}$$

In the matrix formula, *w*_*ij*_=0 when *x*_*j*_ is not the nearest neighbor of *x*_*i*_. Otherwise, the following formula is met as shown below:

(4) $$\begin{array}{c} \underset{j}{\sum }wij=1. \end{array}$$

Locally weighted reconstruction of *k* nearest neighbors of *x*_*j*_ is carried out to obtain the reconstruction matrix as shown in the following formulas:

(5) $$\begin{array}{c} W=\overset{k}{\underset{j=1}{\sum }}\overset{k}{\underset{i=1}{\sum }}wijwilA_{jl}^{i}, \end{array}$$

(6) $$\begin{array}{c} Wij=\frac{\sum_{i=1}^{k}{\left(A_{jl}^{i}\right)}^{-1}}{\sum_{m=1}^{k}\sum_{m=1}^{k}{\left(A_{mn}^{i}\right)}^{-1}}. \end{array}$$

A matrix is a *k* × *k* matrix, as shown in formula (7):

(7) $$\begin{array}{c} {\left(A_{jl}^{i}\right)}^{-1}={\left(xi-xj\right)}^{T}\left(xi-xl\right)\text{.} \end{array}$$

It is called low-dimensional mapping and is used to map all feature vectors of the image to low-dimensional space and keep the original structure of output sample data in low-dimensional space. By locally rebuilding the weight matrix and the feature vectors of its nearest neighbor points of feature vector *x*_*i*_, the output vector *y*_*i*_ of feature vector *x*_*i*_ in low-dimensional embedding space can be calculated. In the process of projection, the cost function is minimized, as shown in formula (8):

(8) $$\begin{array}{c} \Phi \left(Y\right)=Y\left(I-W\right){\left(I-W\right)}^{T}Y^{T}\text{.} \end{array}$$

The Mahalanobis distance in the local linear embedding algorithm is a generalized distance, which is expressed as the distance between a point and a distribution. It is believed that there is a relationship between sample points. The Mahalanobis distance can be defined as shown in formula (9) .

(9) $$\begin{array}{c} d\left(xi,xj\right)=\sqrt{{\left(xi-xj\right)}^{T}\sum^{-1}\left(xi-xj\right)}\text{,} \end{array}$$

where Σ is the covariance matrix of multidimensional random variables. Suppose there are *n* training samples in the data sample set *Q*, then the Mahalanobis distance *d* from sample point *q*_*i*_ to *q*_*j*_ can be expressed as follows:

(10) $$\begin{array}{c} d\left(qi,qj\right)=\sqrt{{\left(qi-qj\right)}^{T}M^{-1}\left(qi-qj\right)}\text{.} \end{array}$$

#### 3.1.4. Recommendation Algorithm for Short Video Content in English Learning

**Figure 5:**
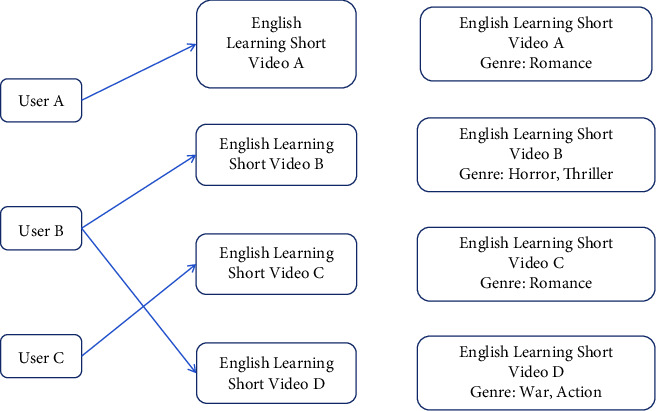
Principles of the short video content recommendation algorithm for English learning.

The recommendation algorithm based on English learning short video content first analyzes the information that the user has browsed before and then analyzes a series of information such as tags, summaries, and auxiliary information. The principle of the content recommendation algorithm for English learning short videos is shown in Figure 5:

The system first needs to model the properties of the item. From the abovementioned analysis, we can find that user A likes English learning short video A and English learning short video A is a romantic video. In addition, we can see that English learning short video C is also a romantic film. Because English learning short video A is the same type as English learning short video C [14]. We guess that user A is also very likely to like short English learning video C. In fact, we usually talk about the type of film, as the name suggests, is based on what story and how to tell the story, so that the type of film has two different concepts. Therefore, according to the content-based recommendation algorithm, we recommend the short English learning video C to user A. In practical applications, less information and insufficient data analysis will appear if the analysis is made only according to the type of articles [15]. It does not work very well, when the training data are insufficient, it is difficult to cook without rice, indicating that the model obtains less information from the original data. In this case, more prior information is needed to ensure the effectiveness of the model. The advantage of a content-based recommendation algorithm is that it does not have the dilemma of cold start of data. According to the attributes of English learning short videos to recommend to users, it can be recommended for people with special interests, and the algorithm based on English learning short video content recommendation has been around for a long time and there are many successful cases in practical applications; the disadvantage is that the feature extraction of English learning short videos is relatively strict. The features of English learning short videos must be able to represent English learning short videos. The key to recommendation is to reflect on its characteristics and the need to conduct an in-depth analysis on it, which will consume a lot of time and energy, but it cannot bring novel things to users, because the recommended English learning short videos are all similar things and the recommendation is more limited [16].

#### 3.1.5. Association Rule Recommendation Algorithm

**Figure 6:**
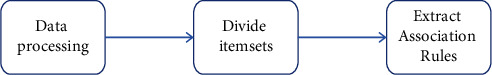
Recommendation process of the association rule recommendation algorithm.

The association rule recommendation algorithm is based on the previous data. If a user buys an English learning short video Y, and another English learning short video Z also appears in the same list of English learning short videos; then, the probability is great. Therefore, the English learning short video Y can be regarded as the premise of this rule, and the English learning short video Z can be regarded as the result of the rule. After purchasing the English learning short video Y, the probability of purchasing the English learning short video Z will also increase. The recommendation process of the association rule recommendation algorithm is shown in Figure 6.

First of all, the input dataset must be standardized and the format is required to be uniform, which is conducive to finding the relationship between the data. Then, the item sets between the data are divided, and the item sets are the collection of short English learning videos jointly purchased by the users. Distance plays an important role in many applications, such as cluster analysis and classification, and different distance measures may get completely opposite results. Because the algorithm needs to analyze the most purchased phase set, finally, the association rules between English learning short videos are analyzed by item sets. Recommendation algorithms for association rules are mainly quantified by support and confidence and recommendation based on association rules is the relationship of different rules according to historical data, and this probability is calculated through historical data. Meaning of support degree: the proportion of English learning short video Y and English learning short video Z appearing in the historical data simultaneously. The meaning of confidence degree: the proportion of data containing English learning short video Z in the data containing English learning short video Y. The advantage of the recommendation algorithm of association rules is that it can find short English learning videos that the user has never contacted for a recommendation, which can surprise users and the algorithm can be operated offline. However, the identification of English learning short video names is a difficulty [17, 18]. The ensemble consensus algorithm has been used for over a decade. As the basis of the consensus algorithm, the ensemble filter algorithm was first proposed by Goldberg et al. in 1992. A collaborative filtering algorithm is first applied in a newsgroup to find the similarity between news and news. Users' interests are divided into long-term interests and short-term interests. The short-term interest may be due to the occurrence of some hot news and long-term interest is the real interest of users. Through users' clicking on news and through this similarity, some interesting information can be pushed to users. In life, if there are users who want to watch short videos of English learning, but they do not know what short videos of English learning to watch for a while. At this time, users may ask their friends, family members, and classmates which English learning short videos are available, and which English learning short videos may be more suitable for the user to watch. When it comes to videos, this generates recommendations. This is also a simple collaborative filtering co-recommendation scenario. Content-based recommendation algorithms recommend users by analyzing the title of the movie, actors, and other information. The principle of a collaborative filtering algorithm is physical clustering, which divides people into groups, gather people with the same interests, and recommends movies they like to each other. The starting point of the collaborative concern recommendation algorithm is that similar users may have a common interest in the same short English learning videos, or the same user may like two similar short English learning videos. Assuming that the videos that users have not seen in the table are represented by Arabic digit 0 and the videos that users have seen are represented by 1, the spatial scores of users *U*_*a*_ and *U*_*b*_ are assumed to be vectors *R*_*a*_, *k* and *R*_*b*_, *k*. The similarity formula between users *a* and *b* can be obtained as shown in formula (11).

(11) $$\begin{array}{c} Sim\left(Ua,Ub\right)=\frac{Ra•Rb}{\left\|Ra\right\|*\left\|Rb\right\|}\text{.} \end{array}$$

**Figure 7:**
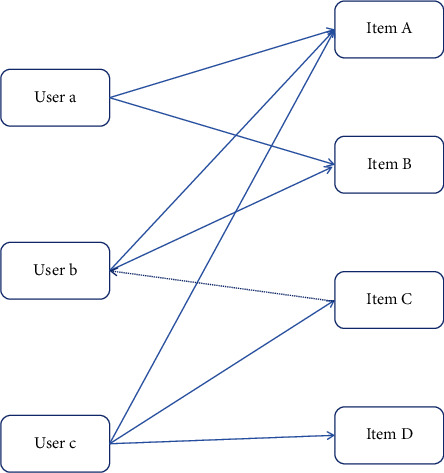
The user collaborative filtering recommendation algorithm.

The user collaborative filtering recommendation algorithm is shown in Figure 7.

The main steps of the user collaborative filtering recommendation algorithm are to find a set of users with similar interests to the target user and to find short English learning videos that the users in the set like and that the target user has not seen before recommending to the target user.

**Figure 8:**
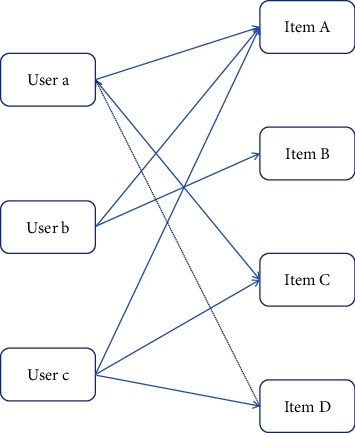
item collaborative filtering recommendation algorithm.

For the recommendation algorithm based on short English learning videos, if many people like two English learning short videos at the same time, the algorithm considers the two English learning short videos to be similar. When users need to recommend, recommend the required short English learning videos to users. If there is no intention or weak intention, it does not need to spend too much time and frequently recommend English videos. It is usually enough to maintain customer relations. The collaborative filtering recommendation algorithm for English learning short videos is shown in Figure 8:

#### 3.1.6. Collaborative Filtering Recommendation Algorithm

A collaborative filtering recommendation algorithm has some advantages: it cannot consider that it is hard to analyze the content-based information automatically. It can be based on a complex or difficult concept to filter. It does not need to model items or users strictly. It does not require a description of the goods that are understandable for the machine. Its recommendation is open and new. It also has some disadvantages: the results of recommendations are influenced by the user's historical preference data. If a user has never reviewed an item, there is no information about that item and it will not be recommended. The core of the recommended method is based on the historical data of previous users; therefore, there is a cold start problem for new users and new items [19].

### 3.2. Time Weighting

#### 3.2.1. Time Factor

Traditional recommendation algorithms only pay attention to the association between users and items, but seldom consider the time factor of users and items. The so-called recommendation system specifically refers to a dynamic system. With the deepening of its understanding, information filtering systems for predicting a user's ratings or preferences for items, relevant researchers find that the time attribute will have a certain impact on the recommendation algorithm (especially its recommendation quality) [20]. As for the users in the system, over time, some old users will leave, while new users will join, so it is recommended that the users in the system are dynamic rather than static. The difference between a dynamic recommendation system and a traditional time-independent static recommendation system is that it can adapt to the changing situation of user interests and item popularity, as well as various time contexts. As for items in the recommendation system, new content may be added, and of course, old and outdated content may also be deleted. Therefore, items, like users, are dynamic and have their own life cycle. Generally speaking, it is obviously of no specific practical value and significance to analyze users' behaviors only while ignoring their specific situations. This is because we cannot analyze all people's behaviors separately from a particular situation. This kind of specific situation also exists in the recommendation system, which is called a context situation. A good recommendation system always recommends to users what they want, allowing users to visit the site more frequently and enhancing user viscosity. For example, user W usually buys some coats in winter, but in summer, the recommendation system still recommends similar coats to the user, which shows that the recommendation system is not intelligent enough to adapt to the situation. Taking another example, when user E is in a very happy mood, he hears cheerful music and gives a high score to this music. However, when the user is in a bad mood, it is obviously inappropriate for the recommendation system to recommend similar cheerful music to the user. Therefore, context information is also a key factor to improve the recommendation quality of the recommendation system. Time, place, and mood are usually contextual information. A short video is a medium of information transmission, which is more timely. When looking for the nearest neighbor, the traditional algorithm may treat all relevant ratings of the user equally, not with the time factor. The playing time of short videos will have an impact on the transmission of videos. The video time is shorter, and the transmission speed is faster. Short videos can attract people's attention and make people like watching them, but users will lose interest in a video over time. For example, user A pays more attention to short videos about cars because he wants to buy a car a few years ago. As time passes and he has bought the car, he loses interest in the short video about the car. So, now is not the time to recommend a short video about a car to user A. Some preferences may change over time. Therefore, the influence of user behavior in the early years on recommendation prediction is relatively small.

#### 3.2.2. Time Weight Calculation

**Figure 9:**
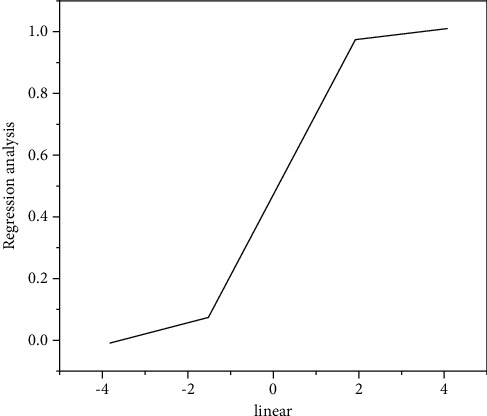
Logistic linear regression analysis model.

In a short time, the user's interest in watching short videos is basically stable. Some preferences may change over time. Based on this problem, it is proposed to add the factor of time into the short video recommendation algorithm based on user behavior analysis. Logistic regression, also known as logistic regression analysis, is a generalized linear regression analysis model widely used in data mining, automatic diagnosis, economic forecasting, and other fields. The logistic linear regression analysis model includes a slow period, logarithmic region, stable region, and a decline period through the correlation method of imitating the growth curve of microorganisms. It can be concluded that the logistic linear regression analysis model is more suitable for the influence of the time factor on short video recommendations. The logistic linear regression analysis model is shown in Figure 9.

The logistic linear regression analysis model function formula is shown in formula (12).

(12) $$\begin{array}{c} \log\;istic\left(ta,j\right)=\frac{1}{1+e^{-ta,j}}\text{.} \end{array}$$

In order to accurately calculate the user's nearest neighbor, the similarity formula of the collaborative filtering recommendation algorithm is shown in the following formula and Algorithm 1.

(13) $$\begin{array}{c} Sim\left(Ua,Ub\right)=\frac{\sum_{ik\in I^{\prime }}\left(Ra,k\times \log\;istic\left(ta,j\right)-\right)\times \left(Rb,k\times \log\;istic\left(ta,j\right)-\right)}{\sqrt{\sum_{ik\in I^{\prime }}{\left(Ra,k\times \log\;istic\left(ta,j\right)-\right)}^{2}}\times \sqrt{\sum_{ik\in I^{\prime }}{\left(Rb,k\times \log\;istic\left(ta,j\right)-\right)}^{2}}}\text{.} \end{array}$$

#### 3.2.3. Algorithm Process

 

### 3.3. Research on the Model of an English Smart Education System

#### 3.3.1. Model Construction

The theoretical basis for the construction of the “double helix” educational system model is the double helix model of biological genetics DNA. This model is formed by two helical curves intertwined with each other. The two curves rotate with each other at the same time, so as to form two spirals and double forces that push and influence each other to generate greater power. College English smart education requires multisubject participation and multielement interaction. The fundamental aim is to make the individual learning needs of learners and the diversified teaching modes of educators form a spiral relationship and to promote the interaction of mutual traction and promotion, and a good cycle is formed between the two. In general, the “double helix” model of English wisdom education adopts the mode of rotation and co-rotation of left and right helix, clockwise, and counterclockwise bidirectional rotation.

In the “double helix” model of English wisdom education, the two helical curves can not only interweave and promote each other but can also rotate in both directions. For example, when a student's personalized self-learning spiral curve rotates too fast or deviates from the main teaching line, the learning spiral curve can be transformed into a reverse rotation mode to adjust students' learning methods and to add more teacher-led traditions of teaching or mentoring. For another example, when the teacher's flipped classroom teaching mode leads to a serious inversion of the tasks before and after class, the spiral of the teachers' diversified teaching mode must be reversed and corrected by rationally allocating the proportion and tasks of extracurricular teaching in the classroom. In this way, the two-way rotation of the spiral is realized, which reflects the flexible adjustment and transformation of the relationship between teaching and learning.

#### 3.3.2. Realization Path of the English Smart Education Model

Smart English education advocates the deep integration of technology and education to meet learners' individual learning needs and complete learners' individual knowledge construction. English classrooms should explore new application modes and methods of new technologies in teaching. The application of new technology tools and platforms such as mobile terminals, the Internet, the Internet of things, and online teaching platforms can better stimulate students' learning enthusiasm, teacher-student interaction, and students' language acquisition and application ability. At the same time, English teachers should construct an open, intelligent, and ubiquitous teaching mode suitable for students of different grades, majors, and learning backgrounds based on their own subject characteristics. In classroom teaching, attention should also be paid to adhering to the teaching concept of teacher-led and student-centered, adopting heuristic teaching, and consciously cultivating students' creativity and imagination. We encourage students to output language in various forms to improve their comprehensive ability to use language. Colleges and universities need to build and improve teachers' informatization learning platforms to improve the informatization learning and practical ability of English teachers. Using advanced scientific and technological means such as the Internet, with the help of new methods such as flipped classrooms and open education classrooms, teachers can conduct online learning, so as to achieve a seamless connection between teachers' work and learning, so as to improve teachers' information literacy. At the same time, it is also strengthening the support and investment of teachers in domestic and foreign training, so that teachers can continuously receive new teaching concepts and learn new teaching skills. In particular, foreign language teachers need to have some experience in studying and visiting abroad. In addition, it cannot be ignored that the entry threshold of college teachers should be raised to improve the overall level and quality of foreign language teachers.

## 4. Experiment and Analysis

The test data used in the experiment were recorded by the research center of the American short video recording system Tubular Labs. The file records contain about 1 million files and 9,343 short video records which were viewed by 1,030 users. Each user can watch nine short videos. The relative speed of the user and video matrix scores is 1000000/(1030 × 9394) = 10.3%. This log file is a small matrix. The initial data are divided into training and testing programs, which account for 80% and 20%, respectively.

This study uses the *F*1 measure method to verify the effectiveness of the time-weighted user behavior analysis recommendation algorithm proposed in this study. The test consists of three metrics, the correct value, the return value, and the *F* value. The classification confusion matrix is shown in Table 1.

The calculation method of the accuracy rate, recall rate, and the F value is shown in formula (14):

(14) $$\begin{array}{c} Accuracy=\frac{TP}{\left(TP+FP\right)}\text{,}\\ recall=\frac{TP}{\left(TP+FN\right)}\text{,}\\ F=\frac{Accuracy\times recall\times 2}{Accuracy+recall}\text{.} \end{array}$$

We take the behavior information of 800 users as the training set of the English learning short video recommendation algorithm and 200 test users as the training set of the recommendation algorithm. In this study, the user behavior analysis English learning short video recommendation algorithm is compared with the common product-based collaborative filtering algorithm. The former focuses on the relationship between users, while the latter focuses on the relationship between English learning short videos. For short English learning short videos, the information itself is less, the quality is different, and the relationship between the videos uploaded by the uploader is disordered, and it is not easy to distinguish, and also it is difficult to find the relationship between the videos. Analyzing the relationship between users is significantly simpler, that is, finding user groups that are similar to users. In this study, the user behavior analysis recommendation algorithm and the uncertain neighbor collaborative filtering algorithm are compared. The uncertain neighbor collaborative filtering algorithm is one of the star algorithms, which balances the relationship between the video and the user by adding an uncertain factor. However, due to the sparse ratings of short videos, it does not fill this data matrix by other means, resulting in a decrease in recommendation quality. The algorithm in this study greatly expands the data matrix by analyzing a large number of users' explicit and invisible behaviors and ensures the quality of recommendation. The user behavior analysis recommendation algorithm in this study is compared with the time-weighted tag collaborative filtering algorithm. It can be seen from the results of both that the time factor has a great influence on the recommendation, so time-weighted is used to improve the quality of the recommendation. The latter focuses on artificially adding tags to videos, which is useful for traditional English learning short videos as it can fully express the characteristics of the English learning short video itself. The number of short videos generated every day is large and the short videos intersect a lot. The shooting staff is not professional and it is difficult to distinguish the types. It is extremely difficult to manually add tags to short videos. The algorithm in this study does not need to consider the internal factors of English learning short videos and directly analyzes the relationship between users. It only needs to find user groups similar to users and the method is simple.

In order to ensure the quality of the initial questionnaire, the reliability of the form is first tested in the research, mainly through the Cronbach *α* coefficient analysis and CITC analysis (Correct Item-Total Correlation), on the basis of which invalid options were eliminated for the questionnaire optimization. Since there are reverse items in this scale, the research first reverse-codes the reverse items. From the Cronbach *α* coefficient, it can be seen that the intelligent reliability coefficient value of the English learning short video recommendation algorithm is 0.884 (>0.8), which has a good reliability level. The intelligent initial question bank of the English learning short video recommendation algorithm is shown in Table 2:

According to the analysis of the CITC value, the corresponding CITC value of “The prediction of the recommended content is not accurate” is less than 0.4. The corresponding CITC value of “I think the recommended content is very homogeneous” is between 0.2 and 0.3, indicating that the correlation between the item and other items is weak. The corresponding CITC value of “The recommended content fails to expand my personal interest” is less than 0.4, and the corresponding CITC value of “Recommended content is often I have seen, lack of freshness” is between 0.2 and 0.3, indicating that the correlation between these four items and other items is weak and can be deleted or modified. According to the exploratory factor analysis, there are multiple cross-factor loads in the initial question. For example, the item “The recommended content can accurately predict my preferences”. It may be ambiguous, which includes the measurement of two dimensions. After the exploratory factor analysis, the factor explanation of the English learning short video recommendation algorithm is still not comprehensive enough, and the content validity is insufficient.

## 5. Conclusions

To sum up, the background of short video recommendations and the research status at home and abroad are analyzed, and it is concluded that short videos will be accompanied by the advent of 5G and it will have more and more economic benefits. It also gives the researchers studying short video recommendation confidence in the future development prospect of English intelligent education based on the short video recommendation algorithm. The original user behavior information is cleaned and normalized. Recommendations are made for the target users by calculating the similarities between each user. The English wisdom education model integrates modern education technology and English teaching. Various modern smart learning tools meet the needs of young students, improve their interest in learning English, and provide students with convenience and conditions for ubiquitous learning. The smart teaching model connects online and offline, enabling students to learn anytime and anywhere, breaking the limitations of traditional classrooms through flipped classrooms, corpus technology, etc., to cultivate students' self-learning ability, big data records students' learning trajectories and provides objective and scientific data and information. Targeted feedback helps teachers adjust their teaching strategies and content promptly, making teaching evidence-based. A variety of intelligent software provide convenience for teaching, but teachers cannot completely rely on tools. English teaching still requires face-to-face communication and interaction between teachers and students.

## Figures and Tables

**Algorithm 1:**
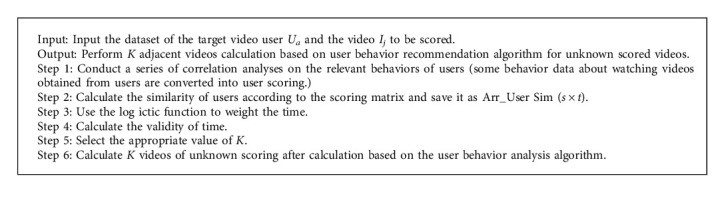


**Table 1:** The classification confusion matrix.

Group	Videos that users actually like	Videos that users actually dislike
Videos recommended by the method	TP	FP
Videos not recommended by the method	FN	TN

**Table 2:** Algorithm recommended intelligent initial question bank.

Group	The content of the question bank
1	The recommendations are all things I really want to see.
2	The recommended content can accurately predict my preferences.
3	Being able to recommend things that I have not seen before and that fit my preferences.
4	The predictions of the recommended content are not accurate.
5	I think the recommendations are rich and varied.
6	The recommended content can cover many aspects of my interests.
7	The recommended content is often different during the same period.
8	I think the recommendations are very homogeneous.
9	The content that does not match my interests are recommended, but I still like it.
10	Recommendation systems can mine my potential likes and dislikes.
11	The recommendations opened up my personal interest.
12	The recommendations fail to expand my personal interests.
13	The recommended content is something I have not seen on other platforms.
14	The recommendations are new and I have been interested in them before.
15	The content seen on the same platform will not be repeated.
16	The recommended content is often what I have seen, which lacks freshness.

## Data Availability

The data supporting the current study are available from the corresponding author upon request.
